# The Hahnemann Club, of Toronto

**Published:** 1888-02

**Authors:** J. D. Tyrrell

**Affiliations:** Secretary and Treasurer


### THE HAHNEMANN CLUB OF TORONTO.

Toronto, December 7th, 1887:—First meeting of the "Hahnemann Club, of Toronto," opened at eight P. M., the President, Dr. Hall, in the chair, and all members present. As this was the *initial* meeting, there is not much to report, save the inaugural address of the President.

### OPENING ADDRESS OF DR. JOHN HALL, SR.

Gentlemen:—The objects of this organization and its tenets, I propose here to speak of briefly.

1. The old society, which usually met monthly, and called itself the "Toronto Homœopathic Medical Society," has ceased to exist, that body having passed a vote that "such society no longer answering the purposes of its framers, be dissolved," which forthwith took effect. Since when, some time last winter, there have been no meetings of medical men calling themselves by our name.

2. The profession of *pure Homœopathy* needed some assembly where the members could openly talk of their doings and experiences.

3. The new organization, as now formed, and calling itself "The Hahnemann Club of Toronto," does not and will not consist of mere "high potency men" (a thing of slow growth and careful diagnosis of the remedy), as our Constitution abundantly shows, but of those who are *true to the Hahnemannian principles;* believing that every curable disease can be managed more *quickly, safely, and thoroughly* by adherence to them than by all other means combined; such cure being always the test of our success. And where a disease is not amenable to medical means, but can only be relieved until death closes the scene, still the homœopathic remedy, the one best agreeing with existing conditions, will often palliate the sufferings of the patient, avoiding the use of Morphine, Chloral, Hydrate, etc. That such agencies as Morphine, etc., may be called for sometimes in such as surgical cases, where the sufferer is almost mashed to pieces in railroad accidents, etc., is, no doubt, true, and to put a sufferer out of his misery, may be desirable. It is, nevertheless, worthy of note, that the *palliative effects of our remedies* are not studied enough. We may also notice poisons as requiring frequently chemical antidotes as their first treatment. The real field of Homœopathy being those diseases, properly so-called, having primarily a dynamic cause, such as small-pox, cholera, scarlatina, measles, croup, diphtheria, puerperal fever, milk leg, carbuncles, and all inflammatory attacks which threaten life quickly; these, one and all, yield in a wonderful manner to our treatment, provided the physician is seen soon after the invasion of such disease. The object, then, which the members of this Club have in view is the treatment of disease of every kind which flesh is heir to, by *true homœopathic means.*

4. Every member is bound, morally, to sustain such an association by every means in his power, and should always have something to communicate out of his own or another's practice, being open to receive as well as give.

5. Every member is also under special obligation to sustain every other member, whether by advice or aid, keeping his moral character also above reproach.

6. The *care* of our medicines should be always with order and neatness, that even our foes may see that we have confidence in what we are doing. As I have no desire that either myself or any one else should give us a long paper, I will close these few remarks here.

Toronto, January 4th, 1888.—Regular meeting of the Hahnemann Club, of Toronto, opened at 8.45 P. M., the President, Dr. Hall, in the chair. After calling of the roll and the usual routine business, Dr. Hall read an instructive paper on "The Causes of Typhoid Fever," as a reply to a similar article appearing a few days before in the *Daily Mail.*

Dr. Eadie called attention to the cure of two cases of tonsillitis, one beginning on right, the other on left side; in the latter a bag-like, œdematous swelling on that side led to the prescription.

Dr. Hall concurred in adaptation of Apis in such cases, when other symptoms, such as œdema and a grayish membrane called for it, and reported a case of diphtheria which began on left side; patient could swallow warm drinks better than cold; Lachesis high; on return he found patient the *same,* with a grayish (blue and white combined), deposit on left side, and no thirst; Apis$^{20m}$, two doses two hours apart, cured in two days. The Doctor also told of a case of diphtheria in which a girl was ill a week before he was called in. His prognosis was great danger of post-diphtheritic paralysis; both tonsils covered with membrane and the only other symptom was the desire *to throw off covering.* Apis high in one week removed all symptoms but paralysis set in, first eyes, then dysphagia, aphonia, and general paralysis, all down spine, so she could not even sit up; dry cough. At intervals of three weeks for each potency, Caust. 6M, 40M, and CM, cured.

Dr. Adams reported Lyc. as curing many cases of herpetic sore throat on right side; Dr. Emory reported an instructive case of hypertrophy of heart, showing the power of the "single remedy" and high potency, even in organic changes. Miss ——, æt. twenty; at five years of age had diphtheria very badly; throat cauterized frequently; for about a year has had much trouble with heart; has been in habit of taking Digit. $\theta$ 10 or 15 gtt., two or three times per day; two or three allopaths diagnosed hypertrophy of heart, and prognosis death in about a year. Careful physical examination found region of superficial cardiac dullness abnormally extended, mostly to left side and upward toward clavicle, also beyond sternum; apex impinges with violence about three-fourths of inch above level of left nipple, midway between it and middle of sternum. Frequent depression; melancholy; often weeps; much dyspnœa, accompanied by palpitation and pain about heart; worse in damp weather, in house, and better in open air; pulse 120; heart beating tumultuously; pulsations visible and *audible clear across the room.* On account of the drugging with Digitalis, gave Nux$^{200}$

April 5th, 1887. April 14th, much better, 11th not nearly so nervous and heart does not beat anything like it did before. Placebo.

May 7th. Has caught cold in head and throat; consults for sores about lips on skin near vermillion border; this has always been the case when she takes cold. The sore patches are round in form, elevated and resemble Herpes circinnatus; from this and history of maltreatment with Arg.-nitr., gave Natr.-mur.$^{200}$. May 12th, pores entirely healed, "not the least bit nervous and heart does not beat at all." Placebo. 28th. Reports that now she never has that violent beating; sleepy after dinner and tired most of time. Natr.-mur.$^{10m}$.

June 18th. Reports feeling quite well now, the heart giving no trouble whatever, even when walking fast or going up-stairs. On careful physical examination I find heart diminished in size fully three-fourths of an inch all around circumference; apex impinging in normal situation. This cure shows what our system can do in organic diseases, and as far as I know adds another to the list of remedies having audible pulsations, if further verified.

It was resolved and adopted that we jointly prepare a monograph on *post-partum and other uterine hemorrhages*; a remedy being assigned to each member to be worked up by our next regular meeting, in February, a fresh remedy each month.

Yours fraternally,

J. D. TYRRELL, *Secretary and Treasurer.*

